# Evaluation of the First Metacarpal Bone Head and Distal Radius Bone Architecture Using Fractal Analysis of Adolescent Hand–Wrist Radiographs

**DOI:** 10.3390/jimaging11030082

**Published:** 2025-03-13

**Authors:** Kader Azlağ Pekince, Adem Pekince

**Affiliations:** 1 Department of Oral and Maxillofacial Radiology, Karabük University, Karabük 78000, Türkiye; 2 Department of Oral and Maxillofacial Radiology, Karabük Oral and Dental Health Education and Research Hospital, Karabük 78000, Türkiye; azlagkader@karabuk.edu.tr

**Keywords:** fractal analysis, bone microarchitecture (or trabecular bone), pubertal development (or skeletal maturation), hand–wrist radiographs, adolescents

## Abstract

The purpose of this study was to investigate changes in bone trabecular structure during adolescence using the fractal analysis (FA) method on hand–wrist radiographs (HWRs) and to evaluate the relationship of these changes with pubertal growth stages. HWRs of healthy individuals aged 8–18 years were included (*N* = 600). Pubertal stages were determined by the Fishman method and divided into 10 groups (early puberty [EP], pre-peak [PRPK], peak [PK], post-peak [PTPK], late puberty [LP]). FA was performed using FIJI (ImageJ) software and the BoneJ plugin on circular regions of interest (ROIs) selected from the first metacarpal bone head and distal radius. Image processing steps were applied according to the White and Rudolph method. Differences between groups were statistically evaluated. Fractal dimension (FD) values of the distal radius (RAFAM) and metacarpal bone head (MAFAM) showed significant differences according to pubertal growth stages (*p* < 0.05). The highest FD value was observed in the LP group, and the lowest FD value was observed in the EP group (except MAFAM in females). FD generally increased from EP to LP in the whole population, but a significant decrease was observed in all groups during the PK period. This decrease was more pronounced in RAFAM of males. These findings suggest a potential decrease of bone mechanical properties in the PK, which is found the be more suitable for orthodontic treatment in the literature. FA on HWRs is a useful and sensitive tool for quantitatively assessing pubertal changes in trabecular bone microarchitecture. The findings demonstrate a significant decrease in FD in both bone regions during the pubertal growth spurt, particularly at the peak period. This may indicate a temporary reduction in bone mechanical strength during this critical stage and could contribute to increased distal radius fracture incidence. Clinically, the relationship between FD and pubertal stages suggests this method could serve as a valuable biomarker in orthodontic treatment planning, allowing for optimized timing of interventions. Furthermore, it may aid in pediatric fracture risk assessment, potentially leading to preventative strategies for high-risk individuals.

## 1. Introduction

The fractal analysis (FA) method is a series of mathematical operations introduced by Mandelbrot that allows the evaluation of complex shapes that cannot be solved by Euclidean mathematics [1].

This method has been widely used in bone research to evaluate bone tissue models, and the results have shown a strong correlation with histomorphometry [2] and bone strength [3] measurements. Fractal geometry has wide application in dentistry, where treatments involving bone tissue are common [4].

As growth continues, particularly during adolescence, the macrostructure of the bone tissue undergoes rapid structural changes, accompanied by a swift adaptation of the internal architecture of the bone to these new conditions. Changes in this process occur due to both biomechanical and hormonal factors [5,6]. Rapid development and growth take place during acceleration periods known as growth spurts, with the preferred growth spurt in orthodontic treatment occurring during adolescence [7]. So, identifying the skeletal maturation period is critical in orthodontic treatment. In the traditional approach, hand–wrist radiographs (HWR) have been employed for this purpose [8]. Nevertheless, fractal dimension (FD) can offer valuable insights into the bone properties in the treated area, thus making it a useful tool in orthodontic treatment [9].

Dual-energy X-ray absorptiometry (DXA), which is the most commonly used method for assessing bone mineral density (BMD) and bone mass or bone mineral content, is considered the gold standard in all age groups, including childhood [10]. The metabolically active nature of trabecular bone, with its greater surface-to-volume ratio, results in a ninefold increase in metabolic activity compared to cortical bone [11]. Consequently, changes in mineralization should be more rapid in trabecular bone than in cortical bone. Accordingly, it is recommended that skeletal development incorporate analysis of bone geometry in addition to bone density or bone mass measurement [5]. Despite studies utilizing DXA, there remains a paucity of clarity in the existing literature regarding the factors that exert the most considerable influence on bone strength. The optimal method for evaluating bone mass in children and adolescents remains a topic of considerable debate in the scientific community [12].

There are numerous reports of fractures increasing during adolescence, particularly in the upper extremities. A study conducted in the USA revealed that pediatric fractures represent a significant proportion of emergency department visits among children. The age group most susceptible to fractures is children between 10 and 14 years old. Forearm fractures are generally the most common type of fracture in children. More than 1 in 4 pediatric fractures involve the distal radius [13,14,15]. Some studies have shown that fractures in this age group are caused by changes in bone trabeculation [16,17]. The physiological justification for this high fracture rate during growth is not clearly explained; however, one of the factors may be the change in bone trabeculation that occurs during peak growth in puberty [18]. In addition, the bone tissue, where the trabecular structure has a complex pattern, is specifically structured in each region of the body and is designed to be able to withstand different forces being exposed to it in different regions. The mechanical properties of bone change not only with the mineral/matrix ratio but also with the micro-scale architectural design of the internal structure [19]. During periods of rapid growth, DXA measurements, typically considered the standard for assessing bone mass, may not always provide accurate results. In 2013, the International Society for Clinical Densitometry (ISCD) reviewed and updated specific guidelines for the interpretation of measurements in children and adolescents to improve the quality and performance of DXA assessments. It has been reported that normal values for bone mass measurements should consider not only age, sex, and ethnicity but also pubertal stage [20].

As dentistry, particularly pedodontics and orthodontics, works mostly on individuals during this period, clinicians are familiar with these changes in their patients and manage some of their treatments according to these skeletal characteristics of the individual. In fact, it has been reported that the success of some orthodontic treatments can be predicted by the FD value calculated in the bone [21].

Additionally, bone cell disorders [22], bone metabolism-related diseases [23], nutritional deficiencies [24], hormonal imbalances [25], and even uremia [26] can negatively impact bone’s mechanical properties, and in these cases, there are many studies that examine bone tissue with FA in the literature. The causal connections between various local and systemic factors impacting bone mass changes and their integration have yet to be thoroughly elucidated, especially in individuals during puberty [27]. It is difficult to monitor and identify the factors that drive bone development during adolescence due to the rapid and complex nature of growth and maturation during this period of life.

This study aims to comprehensively characterize the alterations in trabecular bone microarchitecture during adolescence and their relationship to established pubertal stages as defined by the Fishman method, using fractal analysis (FA) on hand–wrist radiographs (HWRs). The current literature lacks a detailed, quantitative analysis linking these specific microarchitectural changes to defined pubertal milestones; therefore, this research employs a readily accessible and low-radiation imaging approach, going beyond simple age-based assessments to provide a more biologically relevant understanding of bone remodeling. A key focus is the advancement and standardization of FA methodology in pediatric bone research. We utilize a rigorously defined and reproducible protocol for the region of interest (ROI) selection and image processing, adhering to established best practices (e.g., the White and Rudolph method) to minimize bias and ensure comparability with future research. While exploratory, we will also discuss the potential clinical implications of our findings, particularly regarding the optimization of orthodontic treatment timing based on bone remodeling, and the potential link between distal radius trabecular microarchitecture and fracture susceptibility during adolescence.

## 2. Materials and Methods

This study was approved by the Ethical Committee of Karabük University (Date: 2024 No: 1682). The sample consisted of HWRs of healthy individuals between 8 to 18 years of age from the Karabük Oral and Dental Health Training and Research Hospital, Turkey.

This retrospective study utilized hand–wrist radiographs (HWRs) that were originally obtained for routine orthodontic treatment planning and follow-up in a Turkish population. HWRs with positioning errors and artifacts were excluded. A total of 600 radiographs, selected from this existing archive, were included in this study. All HWRs were obtained by the same technician using a panoramic film device (Kodak 8000C Digital Panoramic and Cephalometric System, Cephalostat, Carestream Health Inc., Rochester NY, USA). The images of 30 males and 30 females were selected at random for each stage of puberty, regardless of their age. Sixty images were utilized for each of the 10 pubertal categories examined.

### 2.1. Determination of Pubertal Stages

**Figure 1:**
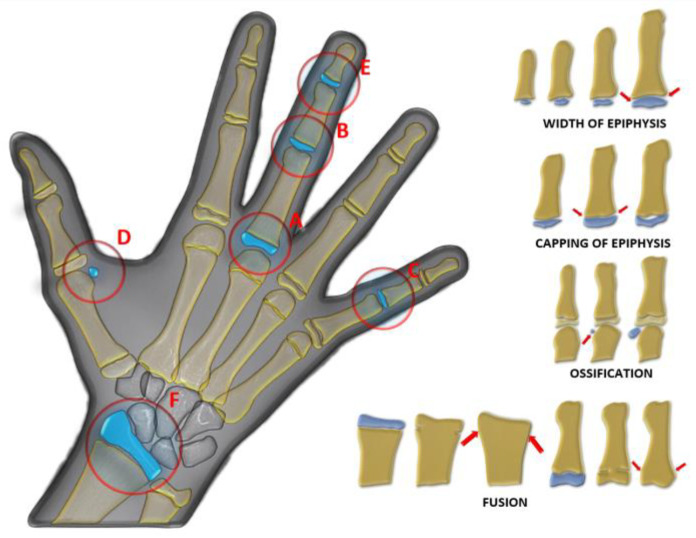
Regions examined (A: proximal phalanx of the third finger, B: middle phalanx of the third finger, C: middle phalanx of the fifth finger, D: adductor sesamoid of the thumb, E: distal phalanx of the third finger, F: distal radius) in hand–wrist radiograph in Fishman classification and epiphysis–diaphysis relationships, diaphyseal equality of the epiphysis (width of epiphysis), epiphysis covering the diaphysis (capping of epiphysis), and ossification and fusion (red arrow).

**Figure 2:**
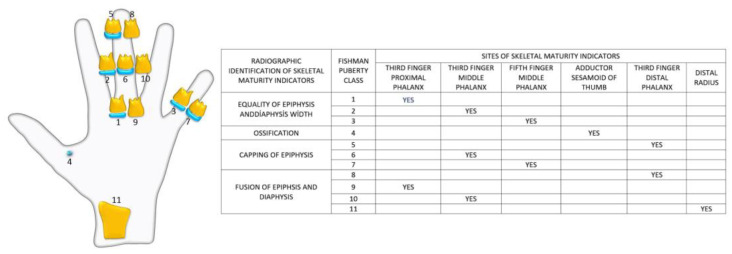
Radiographic identification of skeletal maturity indicators.

The HWRs of 600 patients aged 8–18 years were examined according to the Fishman method, and adolescents were divided into stages [28]. Prior to this classification, 6 regions on the radiographs (proximal phalanx of the third finger (A), middle phalanx of the third finger (B), middle phalanx of the fifth finger (C), adductor sesamoid of the thumb (D), distal phalanx of the third finger (E), distal radius (F)), diaphyseal equality of the epiphysis, epiphysis covering the diaphysis, and ossification and fusion conditions were determined (Figure 1). Afterward, adolescents were classified based on the demonstrated properties. Fishman’s 11th group was excluded because it was not seen in the adolescent growth period and the evaluation was made with the remaining 10 groups (Figure 2). The 10 Fishman adolescent groups were divided into 5 categories: 1–2 early puberty (EP), 3–4 pre-peak (PRPK), 5–6 peak (PK), 7–8 post-peak (PTPK), and 9–10 late puberty (LP). We determined these groups as Pubertal Growth Spurt Classes (PUBC) in accordance with the literature (Figure 2) [29,30,31,32,33].

### 2.2. Fractal Analysis

FIJI, a free image processing application (free ImageJ Fiji software, version 1.54 WS Rasband, NIH, Bethesda, MD, USA) downloaded from the website: “https://ImageJ.net/software/fiji/downloads (accessed on 24 August 2024)”, was used for FA. In the FIJI application, the BoneJ [34] plugin was used for analysis when calculating FD on the final processed images using the box counting method. BoneJ is freely available for anyone to download, use, modify, and distribute. It calculates several trabecular, cross-sectional, and particle parameters in a convenient format. Care has been taken to ensure that measurements are standardized so that results are comparable between studies [35]. To mitigate potential confounding variables arising from disparities in image resolution, all measurements were conducted concurrently on a single laptop device. The specifications of the laptop used for viewing and analyzing the images were as follows: Lenovo T430 2.6-GHz Intel Core i5-3320 M CPU with 4 GB of RAM. The analysis was conducted by a maxillofacial radiologist with over ten years of experience.

### 2.3. Region of Interest Selection

**Figure 3:**
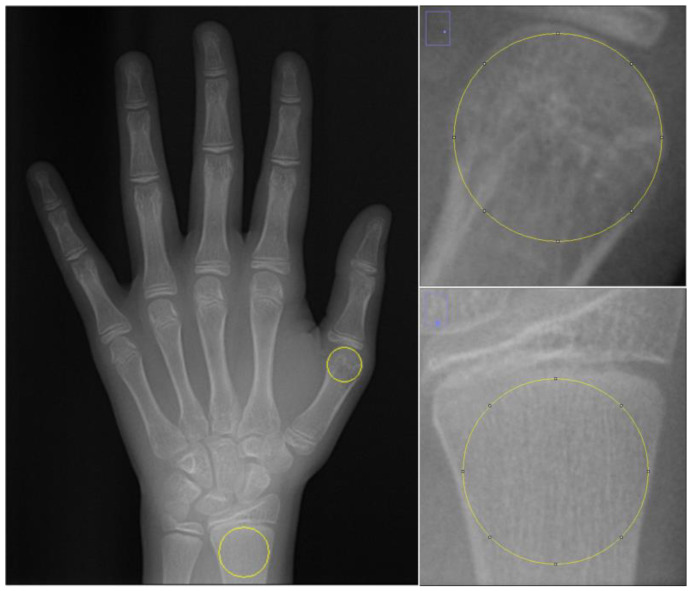
Selection of ROI areas in hand–wrist radiographs.

On the HWR image, a 1495 × 2100 pixel bitmap image (BMP file format), two separate regions of interest (ROIs) were defined: the first metacarpal head and the distal diaphysis of the radius. For each ROI, the largest circle, by area, that could be inscribed within the respective anatomical region was determined. This ensured that each circle was entirely contained within its designated region and did not extend beyond its boundaries. In the first metacarpal, the epiphyseal plate is usually located at the proximal end. Therefore, unlike other metacarpals, the ROI was chosen as the largest circle that can be drawn within the head of the first metacarpal bone, since there is no epiphyseal diaphysis separation in the head of this bone. Since there is an epiphyseal diaphyseal junction line in the distal radius bone, the largest circular area that can be drawn within the region below the epiphyseal diaphyseal junction line was determined as ROI (Figure 3). Circular ROIs were selected as described and duplicated using the tif extension without standardizing their dimensions. To standardize the dimensions of the ROIs, they were all resized to 200 × 200 pixels using the FIJI application. FD was calculated on these ROIs after image processing steps.

### 2.4. The Steps of İmage Processing

**Figure 4:**
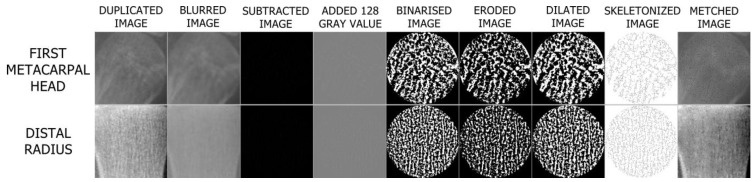
Steps of image processing.

Image processing steps are organized according to the approach of White and Rudolph [36]. ROIs selected and duplicated from the original image were blurred by applying the Gaussian filter with sigma = 4. After subtracting the filtered image from the original image, 128 was added to each pixel value. The image was then binarised. Erosion and dilate operations were performed on the binarised image. The resulting image was finally skeletonized and prepared for FA (Figure 4). FD was calculated by the box counting method using the BONEJ plugin on the FIJI application with automatic parameters that have the smallest box size of 6 pixels, starting box size of 50 pixels, and box scaling factor of 1200.

### 2.5. Statistical Analyses

The data were analyzed using Minitab Statistical Software Version 17.3.1. The compliance of the data to normal distribution was examined using the Kolmogorov–Smirnov test. The FD value calculated at the distal radius (RAFAM) and FD values calculated at the metacarpal bone head (MAFAM) followed a normal distribution when assessed by subgroups, such as Fishman pubertal classes and sex (Table 1). To compare the data, which showed a normal distribution according to PUBC groups, one-way ANOVA was used to determine the difference between groups in the parametric data obtained, and the post hoc Tukey test was used for paired comparisons between groups. To assess intra-observer agreement, the ROIs were reselected in 20% of the images, following the protocol described in the Methods section, and the measurements were repeated. This analysis yielded a kappa index of 0.94 for intra-observer agreement, demonstrating excellent reproducibility. Therefore, statistical analyses were performed on our initial measurements.

## 3. Results

According to the one-way ANOVA test, RAFAM and MAFAM exhibited a statistically significant difference in females, males, and total population in comparison to PUBC. (Table 1 and Table 2).

According to the Tukey pairwise comparison, the LP groups showed that RAFAM was significantly different from all other PUBCs in females, males, and the total sample. MAFAM in the LP group was significantly different for females and overall compared to all other PUBCs. Meanwhile, MAFAM in females showed no significant difference between PK, PRPK, and PTPK groups.

There were no significant differences between the PTPK and PRPK groups in both RAFAM and MAFAM males, and the total population. There was no a statistically significant difference between the PTPK and PRPK groups all other PUBCs, but only in RAFAM for females.

There was a significant difference in the RAFAM values of the PK group and all other PUBCs in the total population. There was no significant difference between the RAFAM values of the PK and PRPK groups in females and the RAFAM values of the PK and EP groups in males. While there was no significant difference between the MAFAM values of only PK and EP in males and in total, there was no significant difference between PK, PRPK, and EP groups in females.

The RAFAM of the EP group in females and in total was significantly different from all other PUBC groups. In males, there was no significant difference between the EP and PK groups’ MAFAM values.

**Graph 1:**
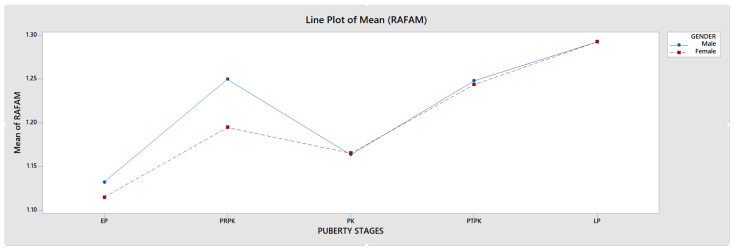
Comparison of RAFAM values in male and female individuals.

**Graph 2:**
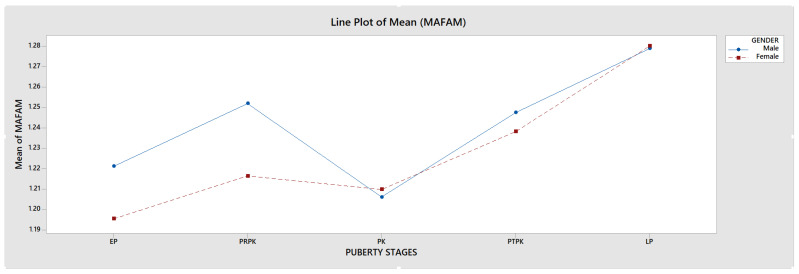
Comparison of MAFAM values in male and female individuals.

The FD value was highest in the LP group in PUBC and lowest in the EP group, excluding MAFAM in females. FD generally increased from EP to LP in the whole population. However, this increase was interrupted by a significant decrease in all groups in the PK period. This decrease in FD was observed more dramatically, especially in the RAFAM values of males (Graph 1 and Graph 2).

If the EP, PRPK, PK, PTPK, and LP classes in PUBC were examined in order of development, there was no significant difference in the transition from PRPK to PK in RAFAM for females. There was a significant difference in all other group transitions in RAFAM females. For RAFAM males, a significant difference was found in all group transitions.

When we examined the group transitions sequentially in MAFAM, no significant difference was found in the transition from PRPK to PK and from PK to PT in females. Significant differences were found in other transitions in females. In males, there was no significant difference in the transition from EP to PRPK and from PTPK to LP, while there was a significant difference in all other transitions.

## 4. Discussion

As chronological age alone is not sufficient to assess development in individuals who are still growing, we believe that a detailed and accurate classification of puberty using different indicators of skeletal maturation in bone tissue examinations of adolescents is also a crucial measure. As skeletal maturation and skeletal age assessed by HWRs are considered the gold standard, they were also preferred in our study [37]. The Fishman method was used to determine pubertal stages, dividing the adolescents into 11 classes and providing a detailed categorization. This approach offered the advantage of observing both puberty and trabecular bone changes on the same radiograph. Of the bone sites we chose for our study, the metaphysis of the distal radius is the most common site of fracture before adulthood and has been extensively studied [38,39,40]. In addition, the distal radius is visible on HWR and was therefore the preferred option in our study.

The first metacarpal is the most active of the hand bones, and unlike the other metacarpals, the epiphysis is located proximally rather than distally. This anatomical condition was advantageous for ROI localization and therefore our selection for the study. Although pseudoepiphysis has been reported in the literature in the area of our metacarpal study, this variant occurs in only 1% of cases, and the incidence of pseudoepiphysis in other metacarpals is much higher [41].

The difference between PUBC was found to be more evident in RAFAM than in MAFAM. We think that this situation occurs because the ROI used in RAFAM also includes the growth plate area in the distal radius. The change in RAFAM compared to PUBC was more evident in males than in females. The fact that the puberty period in females occurs on a wider age scale and over a longer period of time compared to males [28] may explain the observed situation (Table 1).

Skeletal maturation during puberty is the physiological sequence of body changes. The timing of these changes can differ between growing individuals because of differences between their biological clocks. As a result, neither somatic maturation nor chronological age have proven to be reliable indicators of skeletal maturation because of the significant variation in the onset of PUBC [42]. Thus, age differences in PUBCs were not considered statistically in this study.

An accurate assessment of pubertal stage is also essential for determining the potential for facial growth and for choosing the appropriate method and timing of orthodontic treatment. Several studies have shown that radiographic measures of skeletal maturation provide more definitive findings than chronological age for orthodontic treatment because skeletal maturation stages can have a wide, individualized chronological age range [43]. It has been reported that the ideal timing for orthodontic treatment of skeletal disorders is during Fishman’s 4–7 puberty classes, followed by the 1–3, and lastly the 8–11 [44]. This timing is essential for optimal outcomes and should be considered when planning treatment. In our study, we found that FD significantly decreased during periods when orthodontic treatment was reported as more preferable (Graph 1 and Graph 2).

Akbulut et al. conducted a study to assess the efficacy of rapid palatal expansion by evaluating FD calculated in the HWR in two groups of similar average age and found that the FD was significantly lower in the successful group [21]. Pamukcu et al. in their study examined FD on the cervical vertebrae in lateral cephalometric radiographs and reported a significant decrease in C4 compared to pre-pubertal growth spurt values compared to post-pubertal growth spurt values [43]. Gümüş et al. evaluated the condyles, angulus, corpus, and mental region of the mandible with FD during functional orthopedic treatment and found a significant decrease in FD except for the mental region [45]. In our investigation, analogous to the aforementioned studies, we observed a significant statistical reduction in FD within the PK group across the entire population.

The study by Cesur et al. investigated the effects of functional orthopedic appliances on trabecular changes in the mandible using FA [46]. The researchers found a significant difference in FD in the condyle and corpus regions between the treatment group and the control group. Arslan et al. conducted a study analyzing mandibular trabecular changes on panoramic radiographs of patients undergoing reverse headgear therapy and found no statistically significant difference between the groups [47].

Kang et al. explored the midpalatal suture utilizing FA on CBCT images among adolescents and found a negative correlation between FD and age [48]. In 2016, a similar study found a strong correlation between midpalatal suture maturation and FD, according to Kwak et al. [49]. Köse et al. found a negative correlation between FD in the mandibular mental region and total orthodontic treatment time in an FA study on Panoramic Radiography in 2022 [9]. Differences in the findings of the aforementioned studies may be due to factors such as population diversity, type of treatment, and type of image, and the technical parameters of the FA may also affect the result by directly changing the FD.

In FA studies, features such as image extension, resolution, and bit depth affect the FD. In addition, the shape, size, resolution, and localization of the ROI were also considered as parameters that change the FD. Furthermore, FD may change since the technical details in the image processing steps in FA will directly affect the image outputs. Since the same pre-processing processes are applied to both groups in the comparison between groups, some differences in detail are important to ensure standardization in the studies, even if they do not affect the result. In fact, there is limited standardization among studies on FA, and the FD value is not a parameter that can be used to compare studies [4,50,51].

For improved standardization in our study, we were sensitive to the issue of selecting the appropriate ROI. The reason why we chose circular ROIs in the studied regions was that we thought it would be useful to select ROI localization in a more standardized way. In addition, since the anatomical regions examined, especially the metacarpal bone head, are circular rather than square, more bone regions could be detected equally with a circular ROI. If the same ROI size had been used in the studied regions, larger areas would have been detected in individuals with relatively small hands and smaller areas in individuals with large hands. Since the size of the ROI varies from person to person, we resized all ROIs to a similar resolution to avoid this situation so that we could examine exactly the same anatomical area in all individuals at the largest possible size. Choosing a broader ROI width enhances the standardization of ROI localization, as smaller ROI sizes may induce bias in ROI selection [52].

The primary objective of the preprocessing stage in FA studies is to display the trabeculae lines in the skeletonized image, which will be used to calculate the FD. To accomplish this, the WR method favors the Gaussian filter. ImageJ Gaussian filter assumes that out-of-image pixels have a value equal to the nearest edge pixel. This assigns higher weight to edge pixels than to pixels inside the image, and higher weight to corner pixels than to non-corner pixels at the edge. Thus, when smoothing with a very high blur radius, the output will be dominated by the edge pixels and especially the corner pixels. Due to its property, we determined that a sigma value of 4 is the optimal filter parameter for clearly exposing the trabeculae in our study using the Gaussian filter. We show that the original image and skeletonized image can be checked by comparing them with the AND function using the image calculator tool of the ImageJ program, as has been done in the WR method (Figure 4) [36]. We were able to reveal the trabeculae more clearly through this approach utilized in our study.

Due to the complexities of filter parameters and binarization steps in FA studies, Santos et al. proposed different binarization methods in their 2023 study [53]. In some FA studies, an invert operation is performed, which was not present in the original WR method. The invert process may result in FD being calculated on the skeletonized image of the intertrabecular space instead of the trabeculae. Silva et al., in their article published in 2023, mentioned this problem and reported that they achieved a more reliable result by revising the image-processing steps [54]. In our study, inversion was not included, and image processing was performed according to the WR method, which is the most preferred method in FA studies. The box counting method is the most commonly used method for calculating FD, and we have selected it for our study. It is important to appropriately determine the box dimensions in this method, as changes in the box dimensions directly affect the FD. Box sizes exceeding approximately 50% of the image size will cause errors, and the box size should never exceed the total size of the image to meaningfully describe an image. To standardize this parameter, we utilized the BoneJ plug-in’s fractal box count feature. By utilizing the automated parameter option within this plug-in, the selection of box sizes is standardized according to the ROI size.

In our study, we observed a statistically significant alteration in FD in comparison to PUBC. We believe that in this cohort, FD holds potential for utilization in patient follow-up as well as in the planning and timing of bone-related dental treatment. The downward trend observed in FD, especially in the PK period, suggests that orthodontic treatment of skeletal abnormalities will be more appropriate and effective in this period.

There are different methods that can provide important additional information about bone, such as central quantitative computed tomography (central QCT), peripheral QCT (pQCT), high-resolution pQCT (HR-pQCT), bone quantitative ultrasound (QUS), magnetic resonance imaging (MRI), and radiogrammetry [20]. Complexity and error may occur when skeletal growth is not taken into account in tests used to assess bone mass in children and adolescents. It has been reported that some bone measurements with DXA do not give correct results in children of growing age [55]. However, DXA measurements remain the established method for evaluating bone mineral content in both children and adolescents, as is the case with adults [56]. While computed tomography-based techniques (central QCT, pQCT, and HR-pQCT) and MRI have certain benefits in determining actual bone mineral density, they are not yet fully available in clinical practice [57]. Furthermore, central QCT presents drawbacks such as high radiation exposure, while pQCT and HR-pQCT pose difficulties in proper positioning, particularly in children, and also come at a high cost [58]. MRI is costly and challenging to implement in children due to the extended imaging time. Currently, QUS is not a widely used method and lacks formal indication [20].

Around 40% of girls and 50% of boys experience a fracture during childhood and adolescence. The most frequent fracture site among children is the forearm, which accounts for 25–30% of all fractures. The incidence of forearm fractures peaks between the ages of 9–12 years for girls and 11–14 years for boys [59,60]. In our study, RAFAM was found to be at its lowest during the PK stage, particularly among males. This suggests a decline in bone mechanical properties during this stage of puberty. Furthermore, we looked at the mean age in the PRPK and PK periods in our study, which were 11.75 and 13.55 years in boys and 10.92 and 11.79 years in girls (Table 1), aligning with the age range in studies that reported an increased incidence of forearm fractures in this age group.

Nishiyama et al. reported in an HR-pQCT study that the cortical layer of the distal radius bone exhibits greater porosity in males than females during adolescence [61]. In our study, RAFAM was larger in males in the groups before PK. However, this disparity decreased in the groups that underwent PK, including PTPK and LP. It is believed that this discrepancy could be attributed to the earlier onset of puberty in females. Additionally, the images studied highlighted the trabecular arrangement within the bone rather than the bone cortex. Consequently, our study results differ from those of the mentioned study. However, despite the increased cortical bone porosity found in males in the same study, males still demonstrated better fracture resistance as bone mechanical properties are also influenced by trabecular formation. The HR-pQCT study by Kirmani et al. revealed discordant results of cortical porosity and thickness [62] compared to the study conducted by Nishiyama et al. Although both studies used similar methods in the same region, one study employed bone age, while the other utilized Tanner’s puberty classification. This distinction in puberty categorization could potentially impact the results obtained.

Some studies using DXA have found significant increases in bone mass during puberty. However, during the peak period of linear growth, size-adjusted DXA measurements indicate a decrease in areal BMD. Studies of the distal radius with pQCT have not shown changes in trabecular BMD but have shown increases in cortical BMD near the end of adolescence [62]. During the PK period, both RAFAM and MAFAM showed a consistent decrease in our study, similar to size-adjusted DXA measurements (Graph 1 and Graph 2).

Fragility (low-trauma) distal radius fractures are common during adolescence and occur more often in boys than girls. The incidence of distal radius fractures peaks during pubertal growth, and epidemiologic evidence suggests that their incidence has increased over the years. Physical activity and sports account for the majority of fractures. However, changes in activity levels or sports participation do not fully explain the increasing fracture rate [61]. During the peak of the pubertal growth spurt, our study showed a significant reduction in bone trabeculation using FA, possibly indicating a decrease in bone mechanical properties during this time (Graph 1 and Graph 2).

Studies have also shown that there is a decrease in the incidence of distal radius fractures after the peak of pubertal growth [63], and the FD values we obtained in our study show that there is an increase in bone trabeculation in the examined areas after this period (Graph 1 and Graph 2).

Bone outer dimensions may increase during adulthood. This phenomenon has been observed through radiogrammetry by measuring the external diameter of several types of bones. It may be the consequence of an increased endosteal bone resorption with enlargement in the internal diameter. Such a modeling phenomenon would be a response to bone loss, tending to compensate for the reduction in mechanical resistance [64].

FD may be more sensitive than BMD in certain cases, according to reports in the literature. FD has the potential to improve the accuracy of screening for osteoporosis patients as a complementary measure to BMD, as well as to assess the mechanical properties of osteoporotic bone and the reparative effects of bone tissue engineering [65].

No imaging modality can provide a complete assessment of bone health in children and young adults due to their respective limitations. The systematic review and meta-analysis by Shalof et al. 2021 offer strong evidence of a significant correlation between digital radiography (DXR) and DXA in assessing bone health [12]. Using BoneXpert software, DXR measures the bone health index (BHI) on hand radiographs, offering an efficient and objective method to predict fracture risk in children and young adults. The BHI, which measures cortical thickness, length, and width of the three middle metacarpals, was not evaluated in our study since we focused on trabecular changes in the ROIs analyzed by HWR. Furthermore, the BoneXpert program, which is accessed through a paid web interface, is most effective in individuals who are younger than 19 years old and cannot be utilized in those who are older [66]. However, we think that studies in which FA and BHI are evaluated together in HWR are also needed for bone research in this population.

Hands are a typical region where both bone age and pubertal period can be determined. Osteoporotic fractures often occur in the hands, which are a dynamic organ with multiple joints and bones, especially in the distal radius [67]. Research has shown that FA can be used to detect changes in bone trabeculation in the elderly population, particularly in cases of osteoporosis [68].

Dose optimization is important even for low-dose methods as children and adolescents are more susceptible to the risk of radiation-induced biological effects than adults [69]. During a hand–wrist X-ray procedure, the child is exposed to <0.00012 mSv of radiation, thus lower than other daily physiological risks [70]. Furthermore, the HWR method requires an exposure time of less than one second, which is significantly shorter than the minutes-long exposure required by other techniques for bone assessment [71].

Methods like HR-pQCT are highly sensitive to motion artifacts that can happen during imaging, which takes around 2–4 min on average [72]. It is also recognized that there is a substantial rate of repeat radiographs due to motion, particularly in the pediatric population [73]. In addition, if periodic radiographic examinations are needed in a shorter period during adolescence, the number of high-dose examinations that can be performed in a year may need to be limited. Even in the DXA method, which involves a relatively low radiation dose, a serial DXA is recommended after 6–12 months (to monitor disease processes or therapy) to minimize the radiation dose to growing children [55]. HWR scans are simpler, more accessible, and less expensive than other bone measurement methods which has been reported to be especially beneficial in children. HWR is frequently used to assess bone age in disabled children who are more susceptible to low bone quality. Moreover, this type of assessment does not require any additional exposure to ionizing radiation [74].

Several studies demonstrate that the pubertal stage can be determined by assessing ossification in the radius bone alone with HWR [75], and even by assessing ossification in the medial phalanx of the middle finger alone with periapical radiographs [76]. Studies that track trabeculation changes during development by combining FA with these methods are also needed.

Changes in adolescent athletes’ bone structure and pubertal growth timing impact their physical performance [77]. Young athletes are at risk for musculoskeletal injuries specific to the growing skeleton [78]. Similar FA studies can be used to monitor factors that optimize athletes’ athletic development and bone health while preventing injuries.

FD, unlike some other bone measurements, does not have established, universally accepted reference ranges. This presents a limitation, as it is difficult to definitively classify a single FD as “high” or “low” for a specific individual without considering factors like sex, height, body weight, and age, all of which can influence bone dimensions, particularly during puberty. Furthermore, FD alone cannot determine an individual’s pubertal stage. Ideally, reference values incorporating these parameters would be beneficial for interpreting FD results; however, this was beyond the scope of the current study. Despite this limitation, our study demonstrated that FA can effectively track changes in trabecular bone structure during puberty, even in the absence of established reference values. Longitudinal studies, tracking the same individual over time, would likely provide even more detailed and informative results. For such longitudinal follow-up, techniques utilizing intraoral radiographs of a single finger, known for assessing skeletal maturation with a lower radiation dose, may be preferable to serial HWRs. The measurements can be performed on follow-up radiographs of the same individual during puberty. One limitation of this study is that we were unable to evaluate height, weight, and age because we used only one radiograph per individual. More reliable and comparable data can be obtained by analyzing changes in bone trabeculae on radiographs taken during routine dental check-ups. The study is also limited by the absence of comparative data for another bone measurement method. Nevertheless, DXA measurements yield inaccurate results in our target population, and computed tomography-based bone assessment methods may present ethical concerns, particularly in studies involving healthy children due to the relatively high exposure to radiation.

We assert that our study’s strengths lie in the thorough pubertal categorization and standardized FD assessment. Although we calculated the FD in two regions with distinct characteristics, the similarity in the FD pattern across puberty classes indicates the accuracy of our study. In addition, the distal radius region, which is visible in HWRs and was the focus of our examination, has been analyzed with a variety of bone measurement techniques in numerous studies, allowing for the assessment of our findings.

## 5. Conclusions

This study has demonstrated that fractal analysis of adolescent hand–wrist radiographs is a useful and sensitive tool for quantitatively assessing pubertal changes in the trabecular bone microarchitecture of the first metacarpal bone head and distal radius. Our findings reveal a significant decrease in fractal dimension in both bone regions during the pubertal growth spurt, particularly during the peak period. This suggests a potential temporary reduction in bone mechanical strength during this critical developmental stage, which may contribute to the increased incidence of distal radius fractures observed in this age group. The relationship between fractal dimension and pubertal stages suggests that this method could potentially serve as a biomarker in clinical applications, particularly in orthodontic treatment timing and pediatric fracture risk assessment. Future studies should comparatively evaluate fractal analysis results with other bone density measurement methods (e.g., DXA) and biomechanical testing, and investigate the long-term effects of fractal dimension changes on bone health and fracture risk. Furthermore, studies involving larger and more diverse populations will enhance the clinical validity and generalizability of this method.

As a result, it is challenging to assess bone mass in children and adolescents accurately using routine methods. Our study’s data indicate that the FA method can effectively monitor trabecular changes during adolescence.

## Figures and Tables

**Table 1:** Mean RAFAM values, mean MAFAM values, mean chronological age, and age distribution of subjects according to pubertal stages.

	PUBC		Age Distrubution on Pubertal Stages											*N* (%)	Mean Chronological Age (Years) ± SD	RAFAM ± SD	MAFAM ± SD
			8	9	10	11	12	13	14	15	16	17	18				
**Females**	EP		18	14	15	9	4	0	0	0	0	0	0	60 (10%)	9.45 ± 1.254	1.113 ± 0.108	1.193 ± 0.196
	PRPK		0	4	20	21	7	8	0	0	0	0	0	60 (10%)	10.92 ± 1.124	1.195 ± 0.086	1.216 ± 0.069
	PK		0	0	0	24	25	11	0	0	0	0	0	60 (10%)	11.79 ± 0.739	1.165 ± 0.144	1.209 ± 0.121
	PTPK		0	0	1	7	17	25	10	0	0	0	0	60 (10%)	12.60 ± 0.960	1.241 ± 0.154	1.235 ± 0.145
	LP		0	0	1	1	4	24	17	10	2	1	0	60 (10%)	13.64 ± 1.193	1.292 ± 0.113	1.280 ± 0.098
	Total per Age in Females		18	18	37	62	57	68	27	10	2	1	0	300 (50%)			
**Males**	EP		7	12	14	17	9	1	0	0	0	0	0	60 (10%)	10.20 ± 1.299	1.131 ± 0.111	1.220 ± 0.169
	PRPK		0	2	10	14	17	9	8	0	0	0	0	60 (10%)	11.75 ± 1.361	1.249 ± 0.063	1.252 ± 0.114
	PK		0	0	0	0	7	22	22	9	0	0	0	60 (10%)	13.55 ± 0.891	1.163 ± 0.162	1.206 ± 0.118
	PTPK		0	0	0	0	1	15	20	17	7	0	0	60 (10%)	14.23 ± 1.015	1.247 ± 0.153	1.245 ± 0.134
	LP		0	0	0	0	0	3	15	25	12	2	3	60 (10%)	15.06 ± 1.118	1.290 ± 0.125	1.278 ± 0.095
	Total per Age in Males		7	14	24	31	34	50	65	51	19	2	3	300 (50%)			
**Total**	EP		25	26	29	26	13	1	0	0	0	0	0	120 (20%)	9.82 ± 1.326	1.122 ± 0.110	1.206 ± 0.184
	PRPK		0	6	30	35	24	17	8	0	0	0	0	120 (20%)	11.33 ± 1.311	1.222 ± 0.101	1.234 ± 0.100
	PK		0	0	0	24	32	33	22	9	0	0	0	120 (20%)	12.67 ± 1.205	1.164 ± 0.153	1.207 ± 0.110
	PTPK		0	0	1	7	18	40	30	17	7	0	0	120 (20%)	13.42 ± 1.281	1.244 ± 0.135	1.240 ± 0.173
	LP		0	0	1	1	4	27	32	35	14	3	3	120 (20%)	14.35 ± 1.358	1.291 ± 0.119	1.279 ± 0.069
	Total per Age in All		25	32	61	93	91	118	92	61	21	3	3	600 (100%)			

RAFAM, the fractal dimension calculated at the distal radius; MAFAM, the fractal dimension calculated at the metacarpal bone head; EP, early puberty; PRPK, pre-peak; PK, peak; PTPK, post-peak; LP, late puberty; PUBC, Pubertal Growth Spurt Classes; SD, standard deviation.

**Table 2:** Tukey pairwise comparisons and one-way ANOVA test *p*-values according to pubertal stages.

Distal Radius									Metacarpal Bone Head						
Tukey Pairwise Comparisons								One-Way ANOVA	Tukey Pairwise Comparisons		One-Way ANOVA				
	PUBC	*N*	RAFAM Mean FD	Grouping				*p*	PUBC	MAFAM Mean FD	Grouping				*p*
**Females**	LP	60	1.29238	A				0.000 *	LP	1.28032	A				0.000 *
	PTPK	60	1.24128		B				PTPK	1.2356		B			
	PRPK	60	1.19540			C			PRPK	1.21622		B	C		
	PK	60	1.16533			C			PK	1.20897		B	C		
	EP	60	1.11318				D		EP	1.1932			C		
**Males**	LP	60	1.29070	A				0.000 *	LP	1.27822	A				0.000 *
	PRPK	60	1.24930		B				PRPK	1.25203	A	B			
	PTPK	60	1.24733		B				PTPK	1.2459	A	B			
	PK	60	1.1630			C			EP	1.2201		B	C		
	EP	60	1.13127			C			PK	1.20682			C		
**Total**	LP	120	1.29154	A				0.000 *	LP	1.27927	A				0.000 *
	PTPK	120	1.24431		B				PTPK	1.24075		B			
	PRPK	120	1.22235		B				PRPK	1.23413		B			
	PK	120	1.16419			C			PK	1.20789			C		
	EP	120	1.12222				D		EP	1.20665			C		

RAFAM, the fractal dimension calculated at the distal radius; MAFAM, the fractal dimension calculated at the metacarpal bone head; EP, early puberty; PRPK, pre-peak; PK, peak; PTPK, post-peak; LP, late puberty; PUBC, Pubertal Growth Spurt Classes; FD, fractal dimension. Means that do not share a letter are significantly different. * Statistically significant value.

## Data Availability

The original contributions presented in this study are included in this article, and further inquiries can be directed to the corresponding author.
